# Evaluation the Association between Labor Dystocia and Birth Spacing in Iranian Women 

**Published:** 2014-09

**Authors:** Meisam Akhlaghdoust, Nesa Zarbati, Zhila Amirkhani, Sara Naimi, Mohamadreza Sadeghi, Sahar Mohammadi Fateh, Mina Jafarabadi

**Affiliations:** 1 Young Researchers’ Club and Elite, Tehran Medical Branch, Islamic Azad University, Tehran, Iran; 2 Sarem Cell Research Center- SCRC, Sarem Women’s Hospital, Tehran, Iran; 3 Islamic Azad University Research Center, Medical Tehran Branch, Tehran, Iran; 4 Students' Research Committee, Tehran Medical Branch, Islamic Azad University, Tehran, Iran; 5 Reproductive Health Research Center, Tehran University of Medical Sciences, Tehran, Iran

**Keywords:** Labor Dystocia, Inter-Pregnancy Interval, Difficult Childbirth

## Abstract

**Objective:** In view of the effect of hard labor on pregnancy outcomes and to determine risk factors, in this study, the effect of spacing between pregnancies was evaluated for probable effect on the incidence of dystocia in labor.

**Materials and methods:** This is a cross - sectional study used the records of 210 pregnant women referred to Azad University hospitals between January 2000 and December 2012. Dystocia was diagnosed according to ICD-9-CM. Data were analyzed using statistical software Spss_17_.

**Results:** It was found that in pregnancies with 2-4 years spacing between births normal delivery was more prevalent while in the group with 8-10 years spacing labor dystocia was more prevalent.

**Conclusion:** Based on the findings of this study the interval between pregnancies has a significant effect on labor dystocia. Increasing the spacing between pregnancies more than 8 years is a risk factor for dystocia.

## Introduction

Labor dystocia, or difficult childbirth, causes serious mental and physical complications for both mother and baby.

Dystocia derived from the Greek word “tokos” means difficult childbirth (1). Dystocia prevalence is estimated to be 4.8% to 21% among vaginal deliveries and according to the American College of Obstetricians and Gynecologists in 2003, about 60% of cesarean deliveries in the United States are attributable to dystocia diagnosis (2).

More than 70% of maternal deaths are only caused by the five following factors: (i) hemorrhage, (ii) infection, (iii) unsafe abortion, (iv) high blood pressure, and (v) dystocia, which all are considered to be preventable (3). In addition, the following factors are found to be associated with morbidity due to dystocia: birth injuries caused by difficult childbirth, maternal deaths with a prevalence of 8%, increased bleeding during and after delivery, infection of the fetal membranes, rupture of the uterus and vaginal canal, pelvic trauma, and secondary infertility (2, 4, 5).

Furthermore many injuries and neonatal complications caused by difficult childbirth have been shown including various fractures types of skull, clavicle, humerus and femur, glenohumeral joint dislocation, liver and spleen bleedings, nervous system injury and extremity of motion in upper limb , newborn asphyxia and low Apgar scores (3,5,6).

The frequency of dystocia is reported to be influenced by environmental changes and nutritional effects (7). Physical activity status, height, maternal age, number of pregnancies, weight before pregnancy, weight gain during pregnancy, maternal mental state, history of dystocia in individual and family are factors affecting dystocia delivery (2- 6).

Early diagnosis and appropriate treatment in difficult childbirth can guaranty both maternal and fetal health. Some studies show some side effects of improper spacing on maternal and neonatal health, including maternal death risk, third trimester bleeding, anemia, maternal malnutrition, stillbirth and infant mortality (6-8). One way to avoid an unwanted birth is spacing which also balances economical-social development (9). Spacing between pregnancies is an important step in providing maternal and child health. Lack of proper spacing is one of the concerning factors in maternal and child health (7).

There are enormous studies on causes and risks of dystocia. Yet general knowledge of dystocia incidence rate and its relevant causes is still limited. Considering the effect of difficult childbirth on pregnancy outcomes, maternal and neonatal health, and to determine predisposing factors, this study aimed to investigate the effect of spacing between pregnancies as one of the factors influencing the incidence of dystocia delivery in pregnant women admitted to Islamic Azad University Hospitals in a 12-year period.

## Materials and methods

This study is a cross - sectional study using records of pregnant women referred to hospitals affiliated to Tehran Medical Branch, Islamic Azad University, for delivery between January 2000 and December 2012. Inclusion criteria were a history of having only one previous pregnancy, previous vaginal delivery, no record of abortion, identified space between previous and present pregnancy, health confirmation of the reproductive system by the gynecologist. 

The study was approved by research committee of department of gynecology, Tehran Medical Branch, Islamic Azad University. A total of 210 women, G_2_ P_1_ Ab_0_, were divided into the following two groups: (i) group of normal vaginal deliveries (NVD) (n=105) and (ii) group of deliveries with dystocia (n=105). Diagnosis of dystocia in these women was confirmed using International Classification of Diseases, 9 Revision, Clinical Modification (ICD-9-CM). Interval between pregnancies was calculated as the current delivery date to previous delivery date minus current pregnancy gestational age.

The statistical software Spss17 was used for data analysis.

## Results

Total deliveries in this study included 210 pregnant women (105 normal vaginal delivery and 105 with labor dystocia, or difficult childbirth). Two groups were matched regarding the parameters of previous obstetric complications, maternal age, gestational age, maternal BMI, maternal weight gain during pregnancy, birth weight, gender of newborn, history of previous pregnancy and fetal presentation. Among difficult childbirth cases 90.5 % underwent cesarean section and 7 deliveries (6.6%) were done using tools and in 3 cases the deliveries were complicated.

It was found that 200 (95.2%) cases of previous deliveries were uncomplicated deliveries, of which 99 were current difficult childbirth and 101 cases were normal current deliveries. Ten cases (4.8%) had previous complicated deliveries of which 6 cases experienced current difficult childbirth and 4 cases had normal vaginal delivery (P<0.05) (table 1).

The average birth weight was 3189.9 gr and 3166.1 gr in the vaginal delivery group and the group of difficult childbirth respectively (P<0.05). totally 47 boys and 58 girls in natural vaginal deliveries and 57 boys and 48 girls in deliveries associated with dystocia were born (Table 2).

It was found that in women with 2-4 years spacing between their pregnancies normal vaginal delivery and in women with 8-10 years spacing difficult childbirth makes was more common. It was shown that the average BMI in normal vaginal delivery group was 23.45 and in group with difficult delivery was 24.07 (p<0.05). The average weight gain during pregnancy in normal vaginal delivery group was 13.4 kg and in the group with difficult childbirth was 13.35 kg (p<0.05) (Table 2).

Average maternal age was 28 .15± 4.3 (18- 41) years. Average age of mothers in the vaginal delivery group was 27.7 years and in the group of difficult childbirth was 28.5 years (P< 0.05). Gestational age ranged from a minimum of 28 weeks to a maximum of 42 weeks.

**Table 1:** Obstetrical characteristics of second pregnancy in studied women

	**n(%)**	
Vaginal delivery	NVD	105 (50.0)
	Dystocia ( no device)	105 (50.0)
	Total	210 (100.0)
Other routs of delivery	C/S- no complication	95 (45.2)
	Device-no complication	7(3.3)
	with complication	3 (1.4)
	Total	105 (50.0)
Previous labor	no complication	200 (95.2)
	with complication	10 (4.8)
	Total	210 (100.0)
Interval between pregnancies	≤ 2 years	26 (12.4)
	2-4 years	45 (21.4)
	4-6 years	41 (19.5)
	6-8 years	35 ( 16.7)
	8-10 years	31 (14.8)
	More than 10 years	32 (15.2)
	Total	210 (100.0)
infant’s gender	Male	104 (49.5)
	Female	106 (50.5)
	Total	210 (100.0)
Result of previous pregnancy	Preterm labor	10 (4.8)
	IUFD	5 (2.4)
	IUGR	5 (2.4)
	Negative	185 (88.1)
	Fetal death	1 (0.5)
	PROM	1 (0.5)
	LBW	1 (0.5)
	Total	210 (100.0)
Type of Dystocia	Functional	79 (75.2)
	Mechanical	26 (24.8)
	Total	105 (100.0)

Intrauterine fetal death (IUFD)

intrauterine growth restriction (IUGR)

**Table 2:** Comparison of demographic characteristics between two groups.

**Delivery**	**Mean± SD**
Maternal age (year) NVD (n= 105)	27.78± 4.354
Dystocia (n= 105)	28.52± 4.297
Gestational Age (week) NVD (n= 105)	38.9± 1.954
Dystocia (n= 105)	38.79± 2.06
BMI NVD (n= 105)	23.4538± 2.21845
Dystocia (n= 105)	24.0736± 2.74058
Weight gain (kg) NVD (n= 105)	13.0443± 2.69638
Dystocia (n= 105)	13.358± 3.64369
Neonatal weight (gr) NVD (n= 105)	3189.9± 511.686
Dystocia (n= 105)	3166.19± 560.275

The average gestational age at delivery, in group of NVD and in the group with difficult childbirth was 38.9 and 38.79 weeks respectively (P<0.05) (Table 3).

**Table 3:** Obstetrical history characteristics and maternal age in two groups

		**NVD**	**Dystocia**	**Total**
Previous labor	Without complication	101	99	200
	With complication	4	6	10
Interval between pregnancies	<2 years	13	13	26
	2-4 years	28	17	45
	4-6 years	22	19	41
	6-8 years	17	18	35
	8-10 years	11	20	31
	More than 10 years	14	18	32
Neonatal gender	Male	47	57	104
	Female	58	48	106
Result of previous pregnancy	Preterm labor	5	5	10
	IUFD	2	3	5
	IUGR	0	5	5
	Negative	95	90	185
	Fetal death	0	1	1
	PROM	1	0	1
	LBW	0	1	1
Mother age	Under 30 years	80	74	154
	Over 30 years	25	31	56
Total		105	105	210

## Discussion

This study indicated that the spacing between births could be an important factor affecting the labor dystocia. According to the findings, it is concluded that increasing the spacing between pregnancies increases the risk of labor dystocia (p< 0.34).

The optimal distance of 3 to 5 years is recommended between pregnancies (3- 6). According to recommendations of the World Health Organization (2005), a minimum of 24 months is advised to be considered between pregnancies (7). Recent studies published in Columbia University Journal of Medical Association of America in 2010 showed that too short or too long space between pregnancies even causes problems in the birth of second child (1, 2).

It is believed that if the gap between children is less than 18 months or more than 59 months the risk for numerous problems for the mother and children increases (7-9). Also, if the distance between pregnancies is less than 18 months an increased risks of premature birth and low birth weight is expected (1, 2, 10). On the other hand, if the spacing between pregnancies is more than 59 months, the risk of premature birth and low birth weight increases to 6% and 9% respectively (11,12).

Bao-Ping Zhu et al. (2006) expressed that increasing the spacing over 6 years if the maternal age overcomes 35 years results in high-risk pregnancies. In this case, the chance of pregnancy complications such as preeclampsia, spontaneous abortion and dystocia increases (13).

Studies show that there is a direct relationship between spacing and child health. With long spacing between pregnancies even the infant mortality will increase. According to previous studies the mother's body needs rest of at least 2 years after each pregnancy to return to normal situation (1, 2, 13).

The possibility of premature birth and low weight increases if the recovery time between pregnancies is not considered (14).

The general weakness of mother due to previous pregnancy and lactation has effect on the rate of dystocia, prematurity and child’s development (15).

Some researches have showed a statistically significant relationship between height, body mass index and maternal weight gain rate with dystocia deliveries (13, 14). In addition, there is a possible relationship between education, race, religious differences, birth order and seek creation, avoidance of sexual abstinence during breastfeeding and postpartum, with birth spacing (7-9). Other studies have also shown that when marriage age increases, birth spacing decreases, indicating the concern of infertility and genetic disorders. It also means that couples want to reach the desired number of children as soon as possible (16).

According to the results of some studies, the birth spacing between 24 and 48 months has shown to cause dystocia labor, while the best maternal age has been found to be between 20 and 30 years. 

Effect of long or short intervals between pregnancies leading to hard labor could be explained by the following involved factors: increased maternal age, decline in maternal physical health, as well as increased risk of diseases, hormonal disorders and osteoporosis for women approaching menopause. 

Further studies on larger sample sizes are recommended in order to review other predisposing factors for dystocia delivery.

## Conclusion

Based on the findings of this study which is confirmed by other studies it can be inferred that the interval between pregnancies has a significant effect on labor dystocia. Increasing the interval between pregnancies is a risk for dystocia. In singleton births to multiparous mothers, labor dystocia was increased with increment of the interval between deliveries.
